# Dyspnea Due to Laryngeal Cicatricial Pemphigoid: A Case Report

**DOI:** 10.7759/cureus.64495

**Published:** 2024-07-13

**Authors:** Dimitar Pazardzhikliev, Stefan Konsulov, Denis Milkov, Maria Kraeva

**Affiliations:** 1 Department of Otorhinolaryngology, Faculty of Medicine, Medical University of Plovdiv, Plovdiv, BGR; 2 Department of Otorhinolaryngology, University Hospital Kaspela, Plovdiv, BGR; 3 Department of Otorhinolaryngology, St. Cosmas and St. Damian Clinic, Plovdiv, BGR

**Keywords:** supraglottoplasty, high-frequency jet ventilation, mucous membrane pemphigoid, cicatricial pemphigoid, airway stenosis, dyspnea

## Abstract

Cicatricial pemphigoid (CP) is a rare, chronic, vesiculobullous disease characteristically affecting the mucous membranes and healing with cicatrization. Laryngeal involvement is rare and leads to airway stenosis. We present a 74-year-old Caucasian woman with CP, affecting the oral cavity, esophagus, lower eyelids, and larynx. Regardless of regular treatment with hydrocortisone and azathioprine, she developed bilateral cicatrization of the aryepiglottic folds and ovoid stenosis of the laryngeal introitus, leading to dyspnea. To avoid tracheostomy, we were able to utilize infraglottic high-frequency jet ventilation under total intravenous anesthesia to perform a CO2 laser supraglottoplasty with sectioning of the aryepiglottic folds. Post-operatively, her dyspnea at rest resolved; there was no progression at the six- and 12-month follow-up, and she was satisfied with the result.

## Introduction

Cicatricial pemphigoid (CP) is a chronic, vesiculobullous disease with reported annual incidences of 1.16 and 0.87 per million in the French and German populations, respectively [1-3]. It is considered a disease of the elderly, as 94% of patients are older than 50 years, with women being affected almost twice as often as men, without a geographic or racial predilection [4].

Cicatricial pemphigoid was first described by Cooper in 1857 and is histologically similar to bullous pemphigoid with the development of subepidermal bullae, but distinctly, after the bullae rupture, scar tissue develops a unique feature of CP. Another distinction between the two diseases is the predilection of CP to the mucous membranes, in contrast to bullous pemphigoid, which most often affects the skin, giving rise to the introduction of the term “mucous membrane pemphigoid” by Lever in 1953 [4-6]. Presently, the term “cicatricial pemphigoid” is also used, which accurately describes the complications, which are not benign.

The oral mucosa and conjunctivae are the most commonly involved mucosal sites, although any other mucosae may be affected. Oral lesions, which occur in over 90% of cases, may be observed anywhere in the oral cavity, including the buccal mucosa, palate, alveolar ridge, gingivae, tongue, and lips. Additionally, although CP is a disease of the mucous membranes, clinicians should be aware that it affects the skin in 30% of cases [7-8].

Laryngeal involvement occurs in about 20% of cases and is usually in patients with concurrent disease in areas of the upper aerodigestive tract, although cases of isolated laryngeal involvement have been described [8-10]. The most frequent laryngeal localizations are the epiglottis, followed by the vestibule, which appears as erythema and edema of the supraglottis, with widespread vesicles and ulceration leading to subsequent extensive scarring of the larynx [11-12].

## Case presentation

We present the case of a 74-year-old Caucasian woman, whose complaints began with the development of pharyngeal discomfort and pain and the appearance of a single bullous lesion on the epiglottis. She was diagnosed with CP five years later after the subsequent development of numerous oral lesions, and regular treatment with hydrocortisone and azathioprine was started. Additionally, she later developed esophageal stenosis, presenting with significant dysphagia for solid foods. These sequelae of the disease led to a total weight loss of 55 kilograms over one year.

Over the past few months, seven years after the initial disease presentation, the patient began having progressive inspiratory dyspnea, and she walked into the outpatient clinic with dyspnea at rest. Her Karnofsky score was 70%. At admission, she had an erythematous and edematous mucosa of the oral cavity, with numerous erosions bilaterally, as well as adhesion of the buccal mucosa to the gingiva of the mandible on the right. Endoscopically, bilateral cicatrization of the aryepiglottic folds was visualized, leading to ovoid stenosis of the laryngeal introitus. Erosions in the hypopharynx were also present, but there were no pathologic changes in the ventricular and vocal folds, subglottis, or trachea (Video 1). 

**Video 1:** Laryngeal cicatricial pemphigoid with bilateral cicatricization of the aryepiglottic folds.

No other mucous membranes were affected in the patient, except for bilateral cicatrization of the lower eyelids, and she had no skin lesions.

Her prior surgical history includes sclerotherapy and an appendectomy. Concomitant diseases are arterial hypertension, for which she takes Valsartan 160 mg orally in the morning, and spinal enthesopathy. She has no significant risk factors or family history, but she is allergic to streptomycin. She does not smoke tobacco.

Before the surgical procedure, the patient received cardiologic clearance for surgery by a cardiologist, and a single intravenous dose of prophylactic ceftriaxone (2 g) was administered one hour before the surgical procedure. Intravenous propofol and lysthenon were used for general anesthesia induction. Given the airway stenosis and the goal of avoiding tracheostomy, we implemented infraglottic high-frequency jet ventilation through a laser-resistant jet catheter with an outer diameter of 3.8 mm. The oxygen percentage was set to a laser-safe 40% with an inspiration: expiration ratio of 1:2. Total intravenous anesthesia was maintained with propofol, atracurium besylate, and fentanyl.

After sufficient myorelaxation, a Klainsasser laryngoscope was introduced, and using a super-pulsed CO2 laser set to 8.0 W and a micromanipulator, a supraglottoplasty with sectioning of the aryepiglottic folds was performed by an experienced surgeon in laryngeal surgery. No complications were observed during or after the surgical procedure. Post-operatively, her dyspnea at rest resolved, but she had dyspnea on exertion. She was discharged after three days of in-patient observation. The six-month follow-up confirms the preservation of the airway (Video 2). ﻿

**Video 2:** Preserved airway six months after CO2 supraglottoplasty for laryngeal cicatricial pemphigoid.

Her self-reported breathing status is without progression at the 12-month follow-up, and she is satisfied with the result.

The patient gave informed consent for publication, and the Surgical Case REport (SCARE) Checklist, which is a standardized and comprehensive tool for reporting surgical case reports, was used for this case report.

## Discussion

Cicatricial pemphigoid is a chronic autoimmune disease affecting the mucous membranes with the development of vesiculobullous lesions [12]. The antigen most commonly targeted is a hemidesmosomal protein of 180 kD known as bullous pemphigoid antigen 2 (BPAG-2), leading to the development of tense bullae that quickly progress to erosions, characteristically healing with scarring. The disease is marked by linear deposition of immunoglobulin G and complement factor 3 along epithelial basement membranes [7]. Alternatively, immunoglobulin A and immunoglobulin M classes of autoantibodies may be found together with or in place of immunoglobulin G [13].

The sequelae of the disease are specific to the site involved. For example, oral involvement has been described as leading to weight loss due to pain-related impairment of food intake, as observed in our case. Conjunctival involvement may result in blindness, and laryngeal involvement may even be life-threatening, necessitating a tracheostomy [14].

Cicatricial pemphigoid is diagnosed based on the clinical presentation and the tissue and serum detection of autoantibodies. Direct immunofluorescence with the detection of anti-basement membrane zone autoantibodies has the highest sensitivity for the diagnosis of CP, from a 3-4 mm punch biopsy or perilesional excisional biopsy, possibly including a small intact blister. An erosion should not be biopsied. In seropositive CP cases, indirect immunofluorescence on human/primate salt-split skin may be used to detect circulating autoantibodies [15].

An important aspect of the diagnosis is to accurately differentiate other vesiculoulcerative autoimmune diseases such as pemphigus vulgaris, bullous pemphgoid, and epidermolysis bullosa [4]. Our patient had initially presented years ago with a single lesion on the epiglottis, at which point, epiglottitis from other causes would have had to be considered. In regards to laryngeal CP, the differential diagnosis should additionally include diseases presenting with progressive dyspnea, including malignancy.

Cicatricial pemphigoid rarely goes into spontaneous remission, necessitating medicinal treatment [7]. In regard to laryngeal CP, the Third International Consensus on Mucous Membrane Pemphigoid recommends the combination of oral corticosteroids and dapsone as the first-line treatment, while the second-line treatment is high-dose oral tetracyclines. In refractive cases, immunosuppressive agents, mostly mycophenolate mofetil, are additionally recommended [15].

Surgical treatment forms a critical aspect of laryngeal involvement [7]. In the active disease phase and definitively in the past, tracheostomy may have been used to stabilize the airway, as performed by Ito et al. in the case of laryngeal CP with restricted arytenoid movement. Later, after reaching disease remission, more definite treatment is planned. Ito et al. performed a two-stage modified Montgomery’s laryngofissure, treating subsequent cicatricial recurrence with laser therapy [1].

The case of laryngeal CP in a stage of remission that we present was managed only by CO2 laser therapy. Similarly, Chan LS et al. found that CO2 laser therapy is an effective treatment for supraglottic scarring in CP, noting that it may need to be used repeatedly, while the use of adjuvant mitomycin lengthens the symptom-free interval [16]. After a 12-month post-operative follow-up, our patient did not need another CO2 laser intervention, even though we did not utilize mitomycin. Alternatively, Ojha et al. successfully managed a case of laryngeal CP by first debulking the stenosis with cup forceps, followed by a laryngeal microdebrider [1].

The rarity of laryngeal cicatricial pemphigoid must be stressed. Although Lever reported laryngeal involvement in about 20% of cases, Hanson et al., in a series of 142 patients with CP, found that only 13 patients had laryngeal involvement [4,8]. Given that the incidence of CP is about 1 per 1 million people, it can be inferred that the incidence of laryngeal CP is about 1 per 5-10 million people annually [2, 3]. The presented case is unique in that its foremost presentation was laryngeal, a single lesion on the epiglottis, which there is no literature data on incidence.

Laryngeal CP presenting with dyspnea is a therapeutic challenge, and we showed the possibility of avoiding tracheostomy by employing infraglottic high-frequency jet ventilation. Additionally, our case underlines the possibilities of CO2 laser therapy in managing the disease, particularly in the stage of disease remission.

## Conclusions

Cicatricial pemphigoid with laryngeal involvement is rare and primarily treated surgically, especially after the development of dyspnea. Usually treated temporarily or definitively with a tracheostomy, we were able to alternatively utilize infraglottic high-frequency jet ventilation to perform a CO2 laser supraglottoplasty, establishing an airway in a single procedure. The 12-month follow-up of the current case highlights the favorable results of the surgical procedure.
